# Workplace violence, bullying, burnout, job satisfaction and their correlation with depression among Bangladeshi nurses: A cross-sectional survey during the COVID-19 pandemic

**DOI:** 10.1371/journal.pone.0274965

**Published:** 2022-09-22

**Authors:** Saifur Rahman Chowdhury, Humayun Kabir, Sinthia Mazumder, Nahida Akter, Mahmudur Rahman Chowdhury, Ahmed Hossain

**Affiliations:** 1 Department of Public Health, North South University, Dhaka, Bangladesh; 2 Department of Health Research Methods, Evidence and Impact, McMaster University, Hamilton, Ontario, Canada; 3 Penn State Ross and Carol Nese College of Nursing, Penn State University, University Park, PA, United States of America; 4 Begum Rabeya Khatun Chowdhury Nursing College, Sylhet, Bangladesh; 5 School of Medical Sciences, Shahjalal University of Science and Technology, Sylhet, Bangladesh; 6 Health Services Administration, College of Health Sciences, University of Sharjah, Sharjah, United Arab Emirates; Universiti Pertahanan Nasional Malaysia, MALAYSIA

## Abstract

**Background:**

Depression is one of the most serious yet understudied issues among Bangladeshi nurses, bringing health dangers to this workforce. This study aimed to investigate how workplace violence (WPV), bullying, burnout, and job satisfaction are correlated with depression and identify the factors associated with depression among Bangladeshi nurses.

**Methods:**

For this cross-sectional study, data were collected between February 26, 2021, and July 10, 2021 from the Bangladeshi registered nurses. The Workplace Violence Scale (WPVS), the Short Negative Acts Questionnaire [S-NAQ], the Burnout Measure-Short version (BMS), the Short Index of Job Satisfaction (SIJS-5), and the Patient Health Questionnaire (PHQ-9) were used to measure WPV, bullying, burnout, job satisfaction, and depression, respectively. Inferential statistics include Pearson’s correlation test, t-test, one-way ANOVA test, multiple linear regression, and multiple hierarchal regression analyses were performed.

**Results:**

The study investigated 1,264 nurses (70.02% female) with an average age of 28.41 years (SD = 5.54). Depression was positively correlated with WPV, bullying, and burnout and negatively correlated with job satisfaction (p <0.001). According to the multiple linear regression model, depression was significantly lower among nurses with diploma degrees (β = -1.323, 95% CI = -2.149 to -0.497) and bachelor’s degrees (β = -1.327, 95% CI = -2.131 to– 0.523) compared to the nurses with master’s degree. The nurses who worked extended hours (>48 hours) had a significantly higher depression score (β = 1.490, 95% CI = 0.511 to 2.470) than those who worked ≤ 36 hours. Depression was found to be significantly higher among those who did not receive a timely salary (β = 2.136, 95% CI = 1.138 to 3.134), rewards for good works (β = 1.862, 95% CI = 1.117 to 2.607), and who had no training on WPV (β = 0.895, 95% CI = 0.092 to 1.698).

**Conclusions:**

Controlling burnout, bullying, and workplace violence, as well as improving the work environment for nurses and increasing job satisfaction, are the essential indicators of reducing depression. This can be accomplished with integrative support from hospital executives, policymakers, and government officials.

## 1. Introduction

Depression is the most prevalent psychiatric condition with long durations in illness and moderate or severe impacts to function and quality of life in affected individuals [1]. It is characterized by a constant mental state of low mood, a sense of hopelessness, and a loss of interest in activities [2]. According to the World Health Organization (WHO), symptoms of depression can include decreased energy and strength, loss of motivation in daily life activities, feelings of hopelessness or low spirits, poor self-esteem, worthlessness, feeling that life is not worth living, difficulty concentrating, insomnia, and increased or decreased appetite [3]. According to an estimate from WHO, around 300 million people worldwide struggle with depression [4]. Indeed, the number of people suffering from depression surged by 18.4% between 2005 and 2015, according to the WHO [4]. According to a systematic review and meta-analysis, the pooled prevalence of depression in South Asia was 34.1% during the Coronavirus Disease 2019 (COVID-19) [5]. The research found that depression symptoms were influenced by numerous factors such as respondents’ age, education levels, and occupation categories [6].

Frontline nurses faced massive mental health challenges during the COVID-19 pandemic. Since Bangladesh is a lower-middle-income country, healthcare professionals with low-resource settings are highly prone to infection during pandemics [7]. Escalating depression rates among nurses who work in COVID-19 settings are predictable, given that the etiology of COVID-19 is yet unknown, and the consequences are obscure [8]. A study conducted during COVID-19 reported that 50.5% of Bangladeshi nurses suffer from mild to severe depression [9]. Nurses played a critical role in ameliorating COVID-19, despite having a lack of protective equipment, inadequate staffing, and improper safety procedures [2]. Nurses proved their dedication to their profession and patients by putting their lives at risk in emergency rooms, infection control units, critical care, and COVID-19 patient wards during the pandemic who were at risk of developing depression [10].

Workplace violence (WPV) is often lateral, horizontal, or relational aggression and is a significant workplace hazard in global healthcare settings among nurses [11]. Physical assaults and threats of attack directed toward employees are defined as WPV [2]. It can be perpetrated by patients, families, and coworkers [12]. During the COVID-19 outbreak, WPV had a deleterious impact on the mental health of Chinese healthcare personnel. There was an empirically demonstrated link between WPV and psychological issues; for example, those subjected to WPV had a higher prevalence of depression than those not [13]. According to a recent study in Bangladesh, the overall prevalence of WPV among female nurses was 74.46% [14].

Workplace bullying (WPB) is defined as a pattern of offensive behavior by members of an organization, which often exacerbates in intensity with the endeavor to harm [15]. Subsequently, as conditions in the workplace become more erratic, workplace bullying causes considerable psychological damage to nurses [16]. Bullying can cause victims to experience work-related stress, affect individual nurses and quality of care [17]. Research showed that WPB leads to employee turnover, low morale, lower loyalty, declined productivity, and even the current nursing shortage [17, 18]. A cross-sectional study reported poor mental health status across healthcare workers responding to COVID-19, which is highly associated with WPB [19]. Blackstock et al. (2015) identified that workplace bullying had been predicted by informal organizational settings and inefficiency of administrative procedures, which affected the intention to leave the job [15].

Burnout is characterized as a syndrome of emotional weariness, depersonalization, and a sensation of poor personal accomplishment that leads to diminished productivity of employees who work with others [20]. Huo et al. (2021) indicated that during the COVID-19 outbreak, high burnout among Chinese medical personnel was linked to psychological symptoms such as depression. It was reported that burnout levels and moderate depression were significant among nurses who were primarily female, single with bachelor’s degrees, and had experienced for one to ten years [21].

Job satisfaction describes the extent of a person’s contentedness with their job [11]. A study reported that higher levels of depression were associated with lower levels of job satisfaction [22]. A recent study in Canada revealed that lower job satisfaction was related to increased levels of depression and anxiety [23]. However, Bishwajit and colleagues found that nurses’ job satisfaction was enhanced when organizations ensured support and safety at the workplace [24].

The widespread COVID-19 pandemic has resulted in an increase in acts of violence and bullying directed towards healthcare workers (HCWs) [25, 26]. During the COVID-19 outbreak, there had been several reports of acts of violence, harassment, and stigmatization directed toward HCWs as well as patients [27]. It has been reported that these violent actions increased stress levels and, consequently, intensified the psychological effects resulting from moral injuries [28]. In addition, during COVID-19, a significant percentage of HCWs suffered adverse consequences related to their mental health. Studies have demonstrated that nurses have been found to be at a higher risk for WPV and WPB than physicians and other healthcare professionals [29, 30].

However, research found that several job-related and psychological problems such as lower job satisfaction, lower productivity, poor job performance, burnout, depression, and an increased likelihood of employee turnover intent might be caused by workplace bullying and violence [13, 31]. Previous studies investigated the occurrence of bullying and violence and its potential consequences, particularly the relationship between workplace bullying, violence, and symptoms of burnout. Studies showed that workplace bullying could lower job satisfaction and increase burnout [32, 33]. Several studies in many countries reported that WPV reduced nurses’ job satisfaction and performance, as well as impacted patient care negatively, and increased burnout [34, 35]. Based on the previous research, it can be hypothesized that workplace violence and bullying increase burnout level and impact nurses’ job satisfaction, which eventually predict their level of depression.

Bangladesh is a lower-middle-income country where quality healthcare and adequate staffing are insufficient. On the other hand, the COVID-19 pandemic dramatically escalated the workplace demands for nurses. Therefore, it was essential to understand how workplace-related factors such as WPV, bullying, burnout, and job satisfaction were related to Bangladeshi nurses’ depression levels during the COVID-19 pandemic. Moreover, no studies to date investigated the predictors of depression on a broad scale in Bangladesh. So, the present study’s objectives were to assess the correlation of WPV, bullying, burnout, and job satisfaction with depression and identify the factors associated with depression among Bangladeshi clinical nurses.

## 2. Methods

### 2.1 Study design, settings, and population

Between February 26, 2021, and July 10, 2021, during the high wave of COVID-19 outbreak, this cross-sectional study was undertaken among the Bangladeshi nurses available on online platforms and the nurses working in the eight tertiary level hospitals of two large administrative divisions of Bangladesh. These two administrative divisions (Dhaka and Sylhet) were conveniently selected because Dhaka is the capital, and Sylhet is a significant region of Bangladesh. To be eligible to participate in this study, nurses met the following criteria: (1) be a nurse practitioner with the registration of Bangladesh Nurses and Midwifery Council (BNMC), (2) practice in a healthcare setting, (3) be willing to take part, and (4) have a minimum one year of service as a nurse in a healthcare setting. These eligibility criteria were outlined on the front page of the questionnaire, and the nurses who only met the criteria were invited to participate.

### 2.2 Sample size

The required minimum sample size was 1,054 with 80% power, a 95% confidence interval of 0.05 to 1.96, and a 3% margin of error based on 50.5% of Bangladeshi nurses exposed to depression [9]. Finally, from 1,345 obtained responses, 1,264 completed responses were considered in the analysis. Thus, an additional 11% of participants (240) helped to reduce the study’s margin of error.

### 2.3 Data collection questionnaire

Based on the relevant literature review, a semi-structured questionnaire was adapted. Study objectives were explained to the respondents on the front page, and consent to participate was obtained. Sociodemographic variables such as sex, age, marital status, educational level, and monthly salary were considered on the first page of the questionnaire. On the second page of the questionnaire, questions related to occupational characteristics were stated, i.e., type of job, level of hospital, work department, weekly working hours, years of experience, sufficient equipment to manage patients, timely payment of salary, rewards for good job performance, and training to tackle WPV. In the subsequent pages, items regarding the measurement WPV, bullying, burnout, job satisfaction, and depression were stated. One nurse superintendent of a tertiary hospital and one public health expert in Bangladesh reviewed the initial questionnaire. Based on their suggestions, the required modification of the questionnaire was performed. However, before starting data collection, the questionnaire was piloted among 20 nurses, and further modification was done, and these piloted nurses were not included in the final analysis.

### 2.4 Measurements of workplace violence, bullying, burnout, job satisfaction, and depression

#### 2.4.1 Workplace violence (WPV)

The 5-item Workplace Violence Scale (WPVS) was used to assess WPV against nurses [36, 37]. Previous studies, including studies among healthcare professionals, used the WPVS [38, 39], including studies conducted during the COVID-19 pandemic [14]. This scale reports five types of violence: physical attack, mental abuse, threat, verbal sexual harassment, and sexual assault. The frequency of WPV exposure during the previous 12 months was represented by each item’s replies (which varied from 0 to 3). WPVS categorizes each item of WPV exposure frequencies as 0 = never, 1 = once, 2 = twice or three times, and 3 = more than three times. The total score was achieved by adding the scores of each item which ranged from 0 to 15. In this study, the McDonald’s omega reliability coefficient was 0.60, indicating that the scale’s internal consistency is satisfactory.

#### 2.4.2 Workplace bullying (WPB)

We used the 9-item Short Negative Acts Questionnaire (S-NAQ-9) to assess workplace bullying experience [40]. It comprises nine questions that ascertain whether a person was bullied in the preceding six months. The responses ranged from 1 to 5, where 1 denoting "never" and 5 denoting "daily." The overall score on the scale ranges from 9 to 45, with a higher score indicating more bullying. Previous research utilized this scale to evaluate bullying among nurses [41, 42]. In our present study, the 9-item S-NAQ has a McDonald’s omega of 0.88.

#### 2.4.3 Burnout

The Burnout Measure-Short version (BMS-10) was used in this investigation [43]. The BMS-10 is a simple and easy-to-use tool to assess burnout. This instrument measures an individual’s physical, emotional, and mental levels using the critical aspects of burnout thinking in 10 queries. Each item is scored on a seven-point Likert scale, from 1 (never) to 7 (always). Based on the response value of each item, the total response scores across all ten items ranged from 10 to 70. The overall burnout score for an individual was calculated by dividing the total of response values by 10. Therefore, the total burnout score varied from 1 to 7. The BMS-10 was widely used on several populations in numerous studies [43–45]. The McDonald’s omega of 10-item BMS was found to be 0.89.

#### 2.4.4 Job satisfaction

In this study, the nurses’ job satisfaction was assessed using Brayfield and Rothe’s 5-item Short Index of Job Satisfaction (SIJS-5), a self-report psychometric measure [46, 47]. On a five-point scale, 1 represents "strongly disagree," and 5 represents "strongly agree." Consequently, an individual’s job satisfaction level ranged from 5 to 25, with no cut-off level; higher scores indicate greater job satisfaction. A previous study proposed and validated this scale for conducting surveys [48], and this instrument was employed among Jordanian doctors during the COVID-19 pandemic [44]. In this investigation, the internal consistency of this scale was in an acceptable range, with a McDonald’s omega of 0.64.

#### 2.4.5. Depression

The 9-item Patient Health Questionnaire (PHQ-9) was used to determine the depression level among nurses [49,50]. In clinical contexts, this survey instrument is widely used. This instrument has been used several times in Bangladeshi samples, including healthcare professionals [51–53]. Depressed mood, sleeping issues, tiredness, changes in appetite, difficulty concentrating, and thoughts of suicide are all appraised based on the previous two weeks’ symptoms. Items are rated on a four-point Likert scale, with 0 representing "not at all" and 3 representing "nearly every day", and possible scores ranging from 0 to 27, with higher scores representing higher levels of depressive symptoms. McDonald’s omega, the reliability coefficient was 0.82 in this study.

### 2.5 Sample recruitment procedure

**Fig 1:**
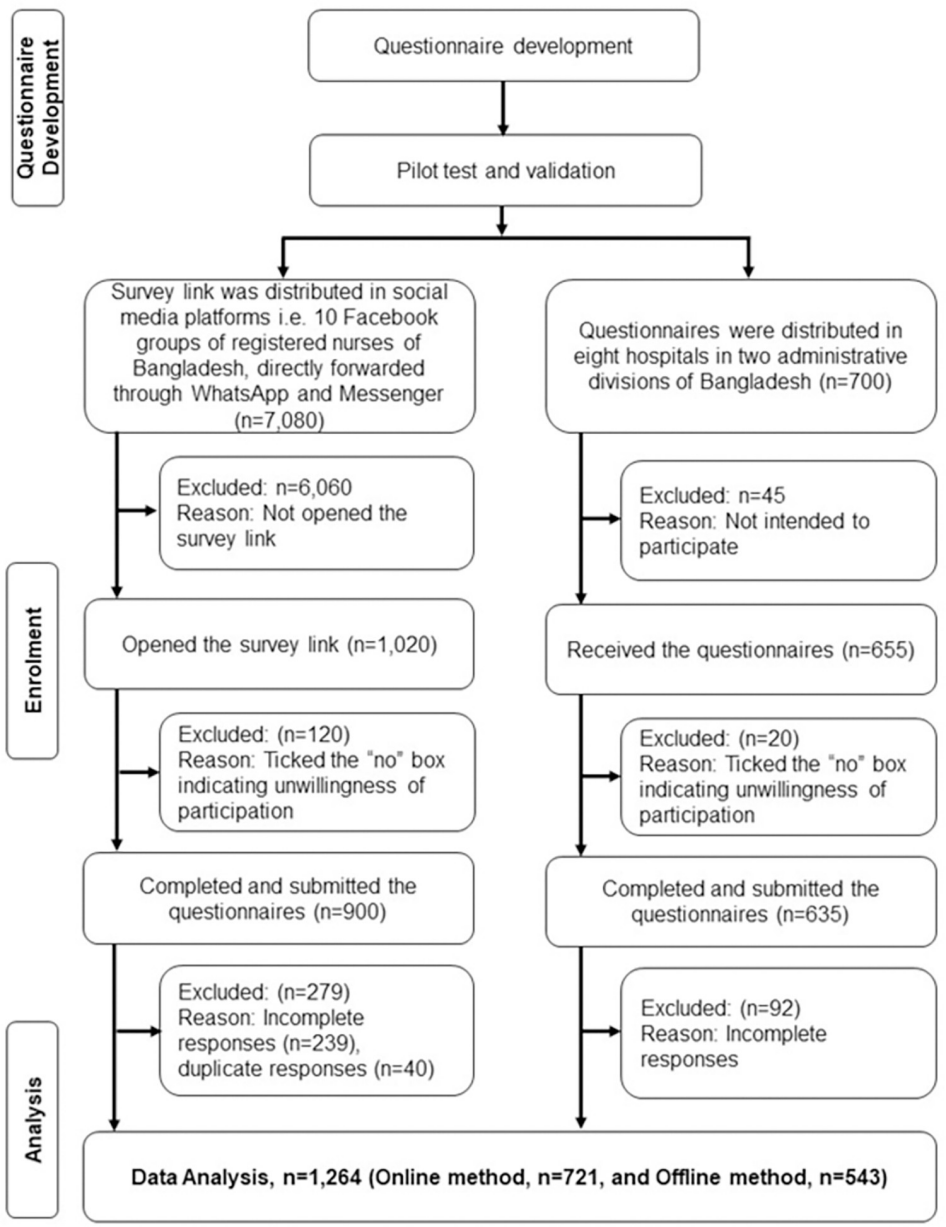
Flowchart of the recruitment process.

In this study, we adopted a non-random convenience sampling technique. Both online (by “Google Form”) and offline (by printed questionnaires) options for data collection were considered. We approached an online method of data collection because face-face interview was restricted during the early stage of the COVID-19 pandemic. Moreover, the online method became a common way of data collection that was adopted by many studies conducted during the early pandemic. We recruited 20 undergraduate nursing students and trained them for data collection. The online questionnaire was developed using "Google Form," and an electronic link was created for data collection. There are various social media platforms, such as Facebook groups, Messenger, and WhatsApp, through which the nurses interact with each other. The trained data collectors posted the questionnaire link daily during the data collection period on these social media sites that only belonged to the nurses. By clicking on the link, nurses were led to the consent form of the questionnaire, and after approving participation in the study, they were taken to the central part of the questionnaire. By online method of data collection, 721 completed responses were obtained. To reach the required sample, another 700 printed questionnaires were distributed to clinical nurses at eight conveniently selected tertiary level hospitals in two large administrative divisions of Bangladesh (Dhaka and Sylhet). The respondents were provided with seven days to complete the questionnaire, and data collectors received questionnaires after seven days. After receiving 655 returned copies, 543 were obtained as completed responses. Thus, a total of 1,264 completed responses were finally included in the current study. To avoid duplication, repeated responses were controlled via ‘Google Form’. Therefore, one respondent could provide only one response. Moreover, we did not distribute printed questionnaires among respondents who participated in online data collection before. The recruitment process of the respondents is presented in the flow diagram (**Fig 1**).

### 2.6 Statistical analysis

The statistical software STATA-16 was used to analyze data. The data yielded from the offline and online collection was entered into an Excel sheet. Finally, the sheet was loaded into STATA-16 for formal analysis. Descriptive statistics such as percentages, frequencies for categorical variables, and mean and standard deviation for continuous variables were performed. Inferential statistics such as t-tests, and one-way ANOVA tests were performed to investigate the mean differences in WPV, bullying, burnout, job satisfaction, and depression scores across demographic and occupational variables. Pearson’s correlation test was performed to assess the correlations among WPV, bullying, burnout, job satisfaction, and depression. A multiple linear regression model was fitted to determine the adjusted association of demographic and occupational variables with depression. The significant variables identified from the t-test and one-way ANOVA test were only included in the modified model. Multiple hierarchical regression models were fitted to investigate the contributory roles of demographic variables, occupational variables, WPV, bullying, burnout, and job satisfaction in predicting depression. All variables were tested for multicollinearity. The current study deemed p <0.05 to be statistically significant.

### 2.7 Ethics statement

On the front page of the questionnaire, the study’s aims and objectives were explained. Before any data was collected, the participants gave their informed written consent after reading about the study’s aims and objectives. The authors ensured that the obtained data remained anonymous and that the respondents were able to withdraw at any time. The Helsinki Declaration 2013 ethical principles for medical research involving human subjects were followed in this study [54]. The ethics committee of Begum Rabeya Khatun Chowdhury Nursing College, Sylhet, Bangladesh (reference number: BRKCNC/IRB/2021/5) gave their formal approval before the study took place.

## 3. Results

### 3.1 Demographic and occupational characteristics of participants (n = 1,264)

**Table 1** describes the demographic and occupational characteristics of the study participants. In this study, 1,264 nurses participated; 70.02% were female. The mean age of the participants was 28.41 (SD = 5.54) years. Nearly half of them were married (53.40%), and 30.14% had a monthly income of BDT 30,000 or above. Among the nurses, 41.14% (520) had diploma degrees, 40.66% (514) had bachelor’s degrees, and 18.2% (230) had master’s degrees or above in nursing. More than half of them (59.81%) worked in the government hospital, and the type of hospital of most of them (72%) was tertiary-level hospitals. The lowest percentage of nurses (18.20%) reported they received WPV training, and were recognized for excellent work performance (20.73%).

**Table 1:** Demographic and occupational characteristics of participants (n = 1,264).

**Demographic variables**	**n**	**Percent/Mean (SD)**
**Sex**		
Male	379	29.98
Female	885	70.02
**Mean age (year)**		28.41 (5.54)
**Age group (year)**		
<25	303	23.97
25–29	604	47.78
≥30	357	28.24
**Marital status**		
Unmarried	589	46.60
Married	675	53.40
**Educational level**		
Diploma degree	520	41.14
Bachelor’s degree	514	40.66
Master’s degree or above	230	18.20
**Monthly salary**		
<21,000 BDT	361	28.86
21,000–29,999 BDT	513	41.01
≥30,000 BDT	377	30.14
**Occupational variables**	**n**	**Percent**
**Type of job**		
Government	756	59.81
Private	508	40.19
**Level of hospital**		
Primary	147	11.63
Secondary	207	16.38
Tertiary	910	71.99
**Work department**		
Critical ward	320	25.32
Emergency	80	6.33
General ward	197	15.59
Gynecological ward	97	7.67
Medicine ward	326	25.79
Surgery ward	244	19.30
**Weekly working hours**		
≤36 hours	597	47.34
37–48 hours	520	41.24
>48 hours	144	11.42
**Years of experience**		
<3 years	435	34.41
3–5 years	404	31.96
≥6 years	425	33.62
**Had sufficient equipment to manage patients**		
Yes	595	47.07
No	669	52.93
**Timely payment of salary**		
Yes	1,134	89.72
No	130	10.28
**Got rewards for good job performance**		
Yes	262	20.73
No	1,002	79.27
**Had training to tackle WPV**		
Yes	230	18.20
No	1,034	81.80

SD = Standard Deviation

n = Number

WPV = Workplace violence

### 3.2 Workplace violence, bullying, burnout, job satisfaction, and depression according to demographic and occupational characteristics

Mean scores of workplace violence (WPV), bullying, burnout, job satisfaction, and depression are presented in **Table 2**. The **Table 3** illustrates the mean differences in WPV, bullying, burnout, job satisfaction, and depression scores across demographic and occupational variables. The nurses who were doing government jobs, the nurses who lacked sufficient equipment, did not get a timely salary, had no rewards for good job performance, and had no previous training to tackle WPV had significantly higher mean scores of WPV (p <0.001). The bullying was found to be considerably higher among the nurses of older age group, married, paid with higher monthly salary, government job holder, highest work experience group, nurses with lack of sufficient equipment for working, nurses not getting a timely salary, nurses with no rewards for good job performance, and nurses with no training to tackle WPV (p <0.001). Similarly, burnout score was significantly higher among the female nurses and nurses with higher educational degrees, lack of sufficient equipment for working, no rewards for good job performance, no training to tackle WPV, and among the nurses who did not receive a timely salary (p <0.001). Job satisfaction score was found to be significantly higher among the higher-income group, government job holders (p <0.001) as well as nurses with a lack of sufficient equipment for working, no rewards for good job performance, no training to tackle WPV, and nurses not getting timely salary (p <0.001). The nurses who were government employees, working more than 48 hours, not getting a timely salary, who did not receive any rewards for job performance, and who had no previous training to tackle WPV had significantly higher mean scores of depression (p <0.001).

**Table 2:** Scores of workplace violence (WPV), bullying, burnout, job satisfaction, and depression.

Scales	Mean±SD	95% CI
Workplace violence	2.77±2.66	2.62 to 2.91
Bullying	17.64±7.29	17.24 to 18.05
Burnout	3.00±1.21	2.94 to 3.07
Job satisfaction	17.30±3.95	17.08 to 17.52
Depression	8.92±5.25	8.63 to 9.21

**Table 3:** Workplace violence (WPV), bullying, burnout, job satisfaction, and depression according to demographic and occupational characteristics.

Demographic variables	WPV	Bullying	Burnout	Job Satisfaction	Depression
	M±SD	M±SD	M±SD	M±SD	M±SD
**Sex**					
Male	2.99±2.84	17.55±7.25	2.78±1.19	17.20±3.85	8.80±5.73
Female	2.67±2.58	17.68±7.31	3.10±1.21	17.35±3.99	8.97±5.04
*F/t*	2.00*	-0.29	-4.33***	-0.58	-0.51
**Age group (year)**					
<25	2.45±2.58	16.80±7.77	3.06±1.18	16.96±3.85	9.21±5.37
25–29	2.84±2.78	17.14±7.24	2.97±1.24	17.32±4.09	8.99±5.35
≥30	2.92±2.52	19.20±6.70	3.02±1.19	17.57±3.78	8.55±4.98
*F/t*	2.99	11.72***	0.61	1.91	1.40
**Marital status**					
Unmarried	2.57±2.63	16.89±7.18	3.01±1.17	16.97±3.84	9.15±5.33
Married	2.94±2.68	18.30±7.32	3.00±1.24	17.59±4.02	8.7±5.19
*F/t*	2.48*	3.43***	-0.22	2.78**	-1.48
**Educational level**					
Diploma degree	2.55±2.61	17.10±7.60	2.87±1.20	17.71±3.92	8.84±5.20
Bachelor’s degree	2.74±2.58	17.96±6.97	3.04±1.18	16.91±3.82	8.66±5.08
Master’s degree or above	3.31±2.89	18.15±7.21	3.23±1.26	17.26±4.21	9.67±5.68
*F/t*	6.62**	2.45	7.34***	5.34**	3.06*
**Monthly salary**					
<21,000 BDT	2.58±2.62	16.68±7.53	3.04±1.27	16.45±3.87	9.55±5.30
21,000–29,999 BDT	2.87±2.72	17.63±7.50	3.02±1.22	17.68±3.94	8.72±5.46
≥30,000 BDT	2.88±2.63	18.73±6.63	2.96±1.13	17.60±3.94	8.59±4.81
*F/t*	1.69	7.33***	0.47	11.91***	3.76*
**Occupational variables**					
**Type of job**					
Government	3.03±2.70	18.37±7.11	3.01±1.18	17.65±3.90	17.65±3.90
Private	2.37±2.56	16.55±7.41	3.00±1.25	16.78±3.96	16.78±3.96
*F/t*	4.36***	4.36***	0.18	3.87***	3.87***
**Level of hospital**					
Primary	2.75±3.02	16.83±7.69	2.98±1.19	17.84±4.38	9.07±5.52
Secondary	3.00±2.80	16.51±6.83	3.04±1.19	17.42±4.03	8.95±5.43
Tertiary	2.72±2.57	18.03±7.29	3.00±1.22	17.19±3.85	8.89±5.18
*F/t*	0.97	4.70**	0.14	1.85	0.08
**Work department**					
Critical ward	2.68±2.55	17.47±7.02	3.06±1.17	17.48±3.85	9.37±5.40
Emergency	3.74±3.13	17.99±6.96	3.09±1.03	17.08±3.37	9.00±5.59
General ward	2.48±2.51	16.84±7.26	3.01±1.21	17.35±4.46	8.95±4.90
Gynecological ward	2.96±2.65	18.91±8.24	3.00±1.31	17.89±4.18	8.70±5.01
Medicine ward	2.91±2.84	17.56±7.67	2.95±1.25	17.51±4.00	9.03±5.38
Surgery ward	2.52±2.45	18.01±6.79	2.97±1.23	16.60±3.57	8.21±5.14
*F/t*	3.83**	1.26	0.39	2.34*	1.43
**Weekly working hours**					
≤36 hours	2.67±2.53	18.03±6.91	3.00±1.23	17.13±3.71	8.53±5.13
37–48 hours	2.76±2.66	17.25±7.76	2.95±1.20	17.53±4.14	8.97±5.20
>48 hours	3.24±3.17	17.56±6.99	3.25±1.12	17.13±4.17	10.43±5.69
*F/t*	2.69	1.57	3.51*	1.54	7.61***
**Years of experience**					
<3 years	2.55±2.67	16.87±7.67	3.07±1.18	17.06±3.87	9.37±5.45
3–5 years	2.77±2.70	17.03±7.15	2.97±1.27	17.23±4.18	8.82±5.48
≥6 years	2.98±2.62	19.02±6.81	2.96±1.18	17.62±3.78	8.55±4.80
*F/t*	2.77	11.57***	1.03	2.21	2.70
**Had sufficient equipment to manage patients**					
Yes	2.19±2.43	15.79±6.17	2.79±1.18	17.82±3.95	8.64±5.10
No	3.28±2.76	19.29±7.80	3.19±1.21	16.84±3.89	9.16±5.38
*F/t*	-7.40***	-8.75***	-5.97***	4.41***	-1.75
**Timely payment of salary**					
Yes	2.66±2.57	17.36±7.04	2.94±1.20	17.51±3.92	8.62±5.12
No	3.71±3.22	20.10±8.79	3.57±1.13	15.51±3.70	11.50±5.67
*F/t*	-4.28***	-4.07***	-5.68***	5.54***	-6.00***
**Got rewards for good job performance**					
Yes	2.14±2.27	16.23±6.32	2.61±1.17	18.16±3.75	7.01±4.56
No	2.93±2.74	18.02±7.48	3.11±1.20	17.08±3.97	9.42±5.31
*F/t*	-4.29***	-3.54***	-5.92***	3.96***	-6.73***
**Had training to tackle WPV**					
Yes	1.90±2.24	15.44±6.96	2.60±1.14	18.25±4.02	7.79±5.12
No	2.96±2.71	18.13±7.27	3.09±1.21	17.09±3.90	9.17±5.25
*F/t*	-5.48***	-5.09***	-5.63***	4.06***	-3.63***

* p < 0.05

** p < 0.01

*** p < 0.001

### 3.3 Pearson’s correlations among workplace violence, bullying, burnout, job satisfaction, and depression

**Table 4** shows the correlations among workplace violence (WPV), bullying, burnout, job satisfaction, and depression. Depression was significantly positively correlated with WPV, bullying, and burnout and negatively correlated with job satisfaction (p <0.001). Similarly, WPV was significantly positively correlated with bullying and burnout and negatively correlated with job satisfaction (p <0.001). Bullying was significantly positively correlated with burnout and negatively correlated with job satisfaction (p <0.001). Burnout was significantly negatively correlated with job satisfaction (p <0.001).

**Table 4:** Pearson’s correlations among workplace violence (WPV), bullying, burnout, job satisfaction, and depression.

Variables	1	2	3	4	5
**Depression**	1				
**Workplace violence**	0.317***	1			
**Bullying**	0.416***	0.493***	1		
**Burnout**	0.605***	0.420***	0.570***	1	
**Job satisfaction**	-0.344***	-0.198***	-0.340***	-0.425***	1

* p < 0.05

** p < 0.01

*** p < 0.001

### 3.4 Demographic and occupational factors associated with depression identified from multiple linear regression model

**Table 5** represents the associated demographic and occupational factors of depression identified from the multiple linear regression model. Compared to the master’s degree holders, depression was significantly lower among nurses with diploma degrees (β = -1.323, 95% CI = -2.149 to -0.497) and bachelor’s degrees (β = -1.327, 95% CI = -2.131 to– 0.523). The nurses who worked more than 48 hours in a week had significantly greater depression (β = 1.490, 95% CI = 0.511 to 2.470) than those who worked ≤ 36 hours. Depression was found to be significantly higher among those who did not receive a timely salary (β = 2.136, 95% CI = 1.138 to 3.134), rewards for good job performance (β = 1.862, 95% CI = 1.117 to 2.607), and who had no training to tackle WPV (β = 0.895, 95% CI = 0.092 to 1.698).

**Table 5:** Demographic and occupational factors associated with depression identified from multiple linear regression model.

Demographic variables	Coefficient, β	95% CI
**Educational level**		
Diploma degree	-1.323**	-2.149 to -0.497
Bachelor’s degree	-1.327**	-2.131 to -0.523
Master’s degree or above		
**Monthly salary**		
<21,000 BDT		
21,000–29,999 BDT	-0.333	-1.300 to 0.634
≥30,000 BDT	-0.333	-1.355 to 0.689
**Occupational variables**		
**Type of job**		
Government		
Private	0.183	-0.794 to 1.160
**Weekly working hours**		
≤36 hours		
37–48 hours	0.145	-0.482 to 0.772
>48 hours	1.490**	0.511 to 2.470
**Years of experience**		
<3 years		
3–5 years	-0.257	-1.009 to 0.495
≥6 years	-0.121	-1.013 to 0.770
**Had sufficient equipment to manage patients**		
Yes		
No	0.186	-0.450 to 0.821
**Timely payment of salary**		
Yes		
No	2.136***	1.138 to 3.134
**Got rewards for good job performance**		
Yes		
No	1.862***	1.117 to 2.607
**Had training to tackle WPV**		
Yes		
No	0.895*	0.092 to 1.698

* p < 0.05

** p < 0.01

*** p < 0.001

### 3.5 Predicting factors of depression

In **Table 6**, the findings from multiple hierarchical regression models are presented. In Block 1, the demographic and occupational variables explained a 6.8% variance of depression. With the addition of WPV in Block 2, explained variance jumped by 7.4% and was fixed at 14.2%. In Block 3, a 9.2% increment was observed after adding bullying, and the total explanatory variance was fixed at 23.3%. The addition of burnout in Block 4 increased the explanatory variance by 15.9%, and the model fixed at 39.3%. Finally, the addition of job satisfaction in Block 5 increased the explanatory variance by 0.8% and explained 40.1% variance of depression.

**Table 6:** Predicting factors of depression.

Variables	Block 1 (β)	Block 2 (β)	Block 3 (β)	Block 4 (β)	Block 5 (β)
Educational level	-0.549**	-0.412*	-0.477*	-0.128	-0.083
Monthly salary	-0.127	-0.081	-0.288	-0.177	-0.127
Type of job	0.248	0.573	0.350	0.120	-0.055
Weekly working hours	0.608**	0.370	0.465*	0.346	0.393*
Years of experience	-0.062	-0.103	-0.237	-0.011	-0.023
Had sufficient equipment to manage patients	0.202	-0.192	-0.682*	-0.817**	-0.903**
Timely payment of salary	2.187***	1.580**	1.082*	0.718	0.661
Got rewards for good job performance	1.773***	1.537***	1.429***	1.016**	1.007**
Had training to tackle WPV	0.923*	0.629	0.555	0.126	0.053
Workplace violence		0.557***	0.240***	0.095	0.097
Bullying			0.255***	0.078***	0.068**
Burnout				2.205***	2.078***
Job satisfaction					-0.126***
F	10.008***	20.355***	33.886***	65.587***	62.409***
R^2^	0.068	0.142	0.233	0.393	0.401
ΔR^2^	0.068***	0.074***	0.092***	0.159***	0.008***

* p < 0.05

** p < 0.01

*** p < 0.001

## 4. Discussion

Working in healthcare settings involves a great deal of physical and mental effort, which can be stressful in many circumstances. Numerous studies have shown that healthcare workers experience depression for a wide range of reasons, and workplace violence (WPV), bullying, burnout, and lack of job satisfaction were identified as significant contributors to depression in nurses. The correlation of WPV, bullying, burnout, and job satisfaction with Bangladeshi nurses’ depression was investigated in this study. This study also investigated the association of demographic and occupational factors with nurses’ depression level.

Our research found a significant correlation between WPV and depression. Similarly, Zafar et al. (2016) found that the professionals who had gone through WPV had higher rates of burnout and were more prone to depression and mental disturbances [55]. According to Fang et al. (2018), psychological distress was detected among Chinese healthcare professionals, but those who experienced physical assault exhibited depressive symptoms [56]. Likewise, another study was conducted among Chinese physicians, which revealed that workplace violence was associated with depression and two measures of burnout (emotional exhaustion and depersonalization) [57].

Workplace bullying was significantly correlated with depression in this study. Our finding is consistent with the finding of Yildirim et al. (2009). They conducted a survey among the Turkish nurses which found workplace bullying led to depression, reduced job enthusiasm, impaired the concentration to care, led to poor performance and lack of dedication to work, and led to poor relationships with patients, supervisors, and coworkers [58]. Another study was conducted among the Norwegian working population and reported that bullying was a significant predictor of outcomes of depression and anxiety, and it was shown to be contributed to the case of turnover intention and absenteeism [41]. Moreover, Sa and Fleming et al. (2008) investigated the occurrence of bullying among Portuguese nurses. They found that bullied nurses experienced much higher levels of psychological tiredness and lower levels of mental wellness [59].

In our study, burnout of the nurses was significantly correlated with their depression. Similarly, burnout was significantly associated with depressive symptoms among Chinese medical workers during the initial COVID-19 surge in China [21]. Hu et al. (2020) examined data of 2,014 frontline nurses in Wuhan, China. They revealed that roughly half of the nurses had moderate to high levels of work burnout, as well as 10.7% of nurses had moderate to high levels of depression [60]. They observed that burnout and depression were statistically negatively correlated with self-efficacy, resilience, social support, and commitment to work in the front lines. Moreover, during COVID-19, the burnout level was high, and a mild to moderate level of depression was also reported among the Turkish frontline nurses [61].

In this study, job satisfaction was negatively correlated with depression. According to Rosa et al. (2021), job dissatisfaction was a significant predictor of depression among nurses during COVID-19, and nurses’ pre-existing mental health conditions were impacted by experiencing a higher number of COVID-19 cases [62]. Similarly, during the COVID-19, among the anesthetists in Rome, a high level of depression, dissatisfaction, and burnout was reported [63]. Further, a study reported that higher job satisfaction was exerted due to greater job control and lower depression among the nurses [64]. Earlier studies among Japanese and Chinese nurses found that job satisfaction and absenteeism were associated with depression and emotional weariness at the same time [65].

Our study also found a significant association of depression with demographic and occupational variables of nurses. In our research, it was found that nurses’ educational level was significantly associated with depression. Nurses with diploma and bachelor’s degrees had less depression than those who completed master’s degrees. On the contrary, a similar survey conducted among nurses in Istanbul, Turkey, found that the nurses who held a higher education degree exerted a lower level of depression [61]. However, Zheng et al. (2021) reported that the nurse’s educational level did not significantly impact depression during COVID-19 [66]. As a second-tier profession in Bangladesh, nursing has less social respect, financial compensation, and work status than other professions [67]. Our findings may explain that nurses with high educational degrees continue to be more prone to psychological oppression considering the social impression, the lack of professional advancement, and the low wage increments available in Bangladesh [9].

According to our study, nurses who worked more than 48 hours per week had higher levels of depression than nurses who worked lesser hours. Considering Bangladesh is a lower-middle-income country, a shortage of nurses obliged them to work longer hours to combat the COVID-19 pandemic. Moreover, due to the shortage of nurses in Bangladesh, some nurses work extra shifts in different hospitals and clinics. As a result, their extended working hours might have an impact on their mental health and elevate their likelihood of being depressed [68]. Geiger-Brown et al. (2006) reported that higher working hours were associated with mental health indicators such as depression, worry, and perceived stress among nursing home assistants in the USA. Doing two or more extra shifts per month was linked to an elevated risk of depression [69]. However, an online-based study on female nurses found that duration of work was not significantly related to the symptoms of depression [70].

Our research found that nurses who were not paid on time had a higher level of depression. In Bangladesh, nursing is considered a "second-tier" career, meaning that it carries a lower job status and offers lower financial pay than other professions [67]. In addition to that, several hospitals and clinics do not pay their nurses on the standard time schedule. Wilson et al. (2020) explained that adverse mental health symptoms were associated with a higher level of economic worry [71]. Similarly, another scientific study pointed out that a salary has long been recognized for its importance in retaining people [72]. Another study recommended focusing on prompt payment to compete for the best and brightest people, boost their job satisfaction, and help them stay less worried about their financial needs, reducing their mental distress [73].

In our study, nurses were asked whether they received any forms of extrinsic rewards as a result of their excellent work performance. According to the findings of our study, the nurses who got rewards for their excellent job performance were less depressed. This finding is supported by Kumari et al. (2021), who emphasized that punishments could be used to deter unwanted behavior while rewards can encourage and foster desirable and constructive behaviors [74]. Nowadays, healthcare organizations operate in a fiercely competitive environment. Thus, the nurses must quickly cope with ever-changing client needs to stay relevant in the job market. However, a reward system from the organizations could be one of the methods for attracting and maintaining qualified personnel and improving their performance.

Our research revealed that the nurses who had no training on WPV were more likely to be depressed. Al-Ali et al. (2016) conducted research among Jordanian nurses to investigate how the training program affected nurses’ attitudes toward workplace violence. The authors found that successful training significantly impacted nurses’ attitudes about reducing work-related mental distress [75]. Similarly, a study explained that nurses’ job satisfaction is linked to innovative organizational initiatives in the workplace [76].

Hospital workers’ and the patients’ overall well-being benefit from determining the causes and possible negative impacts of the problem. As a result, we believe that the findings of our study will assist the relevant authority in ensuring a safe work environment for nurses and secure mental health for them.

### 4.1 Strengths and limitations

This was a comprehensive study to examine how workplace violence (WPV), bullying, burnout, and job satisfaction relate to depression in Bangladeshi nurses. The inclusion of five different instruments in a single study allowed for an extensive analysis of the relationship among these concerns, which are the most important occupational challenges that compromise the quality of care offered by this cohort. Nurses from country’s all administrative divisions were invited to participate in the study, resulting in an optimal sample size. However, there were some limitations of this study as well. Because of using a non-random sampling technique, it was not possible to avoid selection bias. There is a potential for information bias due to using self-reported questionnaire for data collection. Finally, causality could not be established due to the nature of a cross-sectional study.

## 5. Conclusions

Bangladeshi nurses’ depression was correlated to workplace violence, bullying, burnout, and job satisfaction. In addition, the authority’s insufficient professional support predicted nurses’ depression. As a result, hospital administrators, policymakers, and the government must support and implement appropriate policies to address the variables, improve the working environment for nurses, increase job satisfaction, and reduce burnout, bullying, and workplace violence. More in-depth and rigorous investigations on similar difficulties among nurses are needed to establish sustainable work environments.

## Supporting information

S1 Data Dataset of the study can be found in S1 Data file. (XLS)
